# The Beet Cyst Nematode *Heterodera schachtii* Modulates the Expression of WRKY Transcription Factors in Syncytia to Favour Its Development in Arabidopsis Roots

**DOI:** 10.1371/journal.pone.0102360

**Published:** 2014-07-17

**Authors:** Muhammad Amjad Ali, Krzysztof Wieczorek, David P. Kreil, Holger Bohlmann

**Affiliations:** 1 Division of Plant Protection, Department of Crop Sciences, University of Natural Resources and Life Sciences, Vienna, Austria; 2 Department of Plant Pathology, Centre of Agricultural Biochemistry and Biotechnology (CABB), University of Agriculture, Faisalabad, Pakistan; 3 Chair of Bioinformatics, Department of Biotechnology, University of Natural Resources and Life Sciences, Vienna, Austria; 4 School of Life Sciences, University of Warwick, Coventry, United Kingdom; James Hutton Institute, United Kingdom

## Abstract

Cyst nematodes invade the roots of their host plants as second stage juveniles and induce a syncytium which is the only source of nutrients throughout their life. A recent transcriptome analysis of syncytia induced by the beet cyst nematode *Heterodera schachtii* in Arabidopsis roots has shown that thousands of genes are up-regulated or down-regulated in syncytia as compared to root segments from uninfected plants. Among the down-regulated genes are many which code for WRKY transcription factors. Arabidopsis contains 66 *WRKY* genes with 59 represented by the ATH1 GeneChip. Of these, 28 were significantly down-regulated and 6 up-regulated in syncytia as compared to control root segments. We have studied here the down-regulated genes *WRKY6*, *WRKY11*, *WRKY17* and *WRKY33* in detail. We confirmed the down-regulation in syncytia with promoter::GUS lines. Using various overexpression lines and mutants it was shown that the down-regulation of these WRKY genes is important for nematode development, probably through interfering with plant defense reactions. In case of *WRKY33*, this might involve the production of the phytoalexin camalexin.

## Introduction

Plant parasitic nematodes are obligate biotrophic parasites of a large number of plant species. Many of them have a wide host range and can have adverse effects on a variety of important crop plants, either directly or as virus vectors. The worldwide annual crop losses caused by plant parasitic nematodes have been estimated at 157 billion dollars [1]. Cyst nematodes are very important plant parasitic nematodes which induce specialized feeding structures in the infected plant roots called syncytia, the sole nutrient source for the development of these nematodes [2].

The sugar beet cyst nematode *Heterodera schachtii* can complete its life cycle on Arabidopsis plants *in vitro* within 6 weeks and this model system is widely used for studying plant nematode interactions at the molecular level [3]. By using this model system, we recently performed a transcriptome analysis of 5 and 15 dpi (days post inoculation) syncytia using Affymetrix GeneChip analysis with microaspirated syncytial material [4]. This study has revealed that 34.2% out of a total of 21,138 Arabidopsis genes were differentially expressed as compared to uninfected control root segments. Out of these differentially expressed genes, 18.4% (3893) were up-regulated while 15.8% (3338) were down-regulated [4]. This and other transcriptome studies conducted on different plant species have demonstrated high metabolic activity in the nematode feeding cells [4], [5], [6], [7], [8], [9].

In addition to genes related to metabolic activity, many other genes were found to be strongly up-regulated in the *H. schachtii* interaction with Arabidopsis. These genes included those which code for proteins which are involved in cell wall degradation such as expansins, cellulases, and pectate lyases [10], [11], [12] and genes coding for myo-inositol oxygenases [13] and an AAA^+^ATPase [14]. On the other hand, genes which were strongly repressed after nematode infection were related to defense responses of the plant [4] and we have recently shown that one of these strongly downregulated genes, *AtRAP2.6*, is involved in the resistance against *H. schachtii* [15]. Furthermore, one strongly down-regulated group of genes coded for peroxidases and out of 100 differentially expressed genes with the strongest downregulation, 14 were peroxidases [4].

Similarly, we noted that many members of another gene family, which codes for DNA binding WRKY transcription factors, were also significantly down-regulated [4]. The WRKY transcription factors are one of the largest gene families for transcriptional regulators in plants and form essential parts of different signaling pathways that modulate many plant processes, including plant defense (reviewed by [16], [17], [18]. The WRKY transcription factors are characterized by a highly conserved signature domain, WRKYGQK, which corresponds to the most N-terminal β-strand. It has been proposed that this WRKYGQK motif protrudes from the surface of the protein to access the major DNA groove and contact an approximately 6-bp region (TTGACC/T) known as the W box [19].

WRKY transcription factors are involved in a variety of plant processes including biotic and abiotic stresses, seed development, germination and dormancy, senescence, and plant development (reviewed by [16]. Many of the previous reports concerning WRKY transcription factors demonstrated the involvement of different members of this multigene family in the transcriptional reprogramming linked to plant defense responses. WRKY factors are main players of the innate immune system of plants [17]. In these networks, many WRKY proteins interact with MAP kinases [20].

Up to now, only one WRKY gene has been found that was involved in the interaction of nematodes and plant roots. Expression of *WRKY23* was up-regulated in syncytia induced by *H. schachtii* and giant cells induced by *Meloidogyne incognita*, respectively, in Arabidopsis roots [21]. The authors confirmed the up-regulation with GUS lines and *in situ* RT-PCR. Moreover, knock-down of the gene resulted in lower susceptibility against *H. schachtii*, indicating the importance of this gene for nematode development.

The transcriptome analysis of syncytia showed that *WRKY33* was the most strongly down-regulated *WRKY* gene in syncytia as compared to uninfected roots [4]. Among different WRKY proteins in Arabidopsis, WRKY33 has been intensively studied in response to biotic and abiotic stresses. It has been shown that WRKY33 is an important regulator of the genes involved in synthesis of the antimicrobial compound camalexin [26] and ethylene biosynthesis [46]. Recently, it was reported that MPK4 and its substrate MKS1 interact with WRKY33 *in vivo*, and that WRKY33 is released from complexes with MPK4 upon infection. Similarly, transcriptome analysis of a *wrky33* loss-of-function mutant recognized a subset of defense-related genes (i.e. *PAD3*/*CYP71B15* and *CYP71A13*, involved in the production of camalexin) as putative targets of WRKY33 [22], [23], [24], [25]. WRKY33 itself is phosphorylated by MPK3/MPK6 which then leads to the expression of camalexin biosynthetic genes and camalexin production [26]. WRKY33 has also been shown to be important for resistance against the necrotrophic fungal pathogen *Botrytis cinerea* [27] and both, WRKY25 and WRKY33, are important for tolerance to salt stress in Arabidopsis [28]. The *AtWRKY33* promoter contains a set of three WRKY- specific *cis*-acting DNA elements (W boxes) [29] and it was recently demonstrated that WRKY33 binds its own promoter *in vivo* which might lead to a positive feedback loop [26].

Other genes that were strongly down-regulated in syncytia included genes *WRKY6*, *WRKY11, and WRKY17.* The Arabidopsis *WRKY6* gene was expressed in senescent leaves and induced through SA, JA, ethylene and flagellin22 [30]. *WRKY6* positively influenced the *PR1* gene: A *PR1*::*GUS* construct was strongly expressed in senescent leaves and, although at a lower level, in leaves overexpressing *WRKY6*. RT-PCR showed that in addition to *PR1* also *NPR1* was up-regulated in leaves overexpressing *WRKY6* [32]. *NPR1* can be activated by WRKY factors via W-boxes present in its promoter [31]. From these data the authors concluded that *WRKY6* induces *PR1* expression indirectly via *NPR1* [32].

On the other hand, *WRKY11* and *WRKY17* have been reported to act as negative regulators of basal resistance to the bacterial pathogen *Pseudomonas syringae* (Journot-Catalino et al. 2006). Since *WRKY33*, *WRKY6*, *WRKY11*, and *WRKY17* were among the most strongly down-regulated genes in syncytia [4] we have studied these genes in more detail. We reasoned that these genes might be suppressed by *H. schachtii* to avoid a plant resistance response. We have therefore tested if overexpression or knocking out of these genes might have an effect on the susceptibility of Arabidopsis plants to *H. schachtii*.

## Materials and Methods

### Plant cultivation

Arabidopsis ecotype Columbia (Col) plants were grown in soil in a growth room at 25°C in long day conditions (16 h light/8 hour dark). For growth in sterile conditions, seeds were surface sterilized for 7 min in 10% (w/v) sodium hypochlorite. They were then washed three times with sterile water and placed in Petri dishes (9 cm) on either a modified Knop medium with 2% sucrose [3] or on MS medium containing 3% sucrose [33].

### Overexpression, promoter::GUS fusion and mutant lines

The promoter::GUS and overexpression constructs were introduced into *Agrobacterium tumefaciens* GV3101 for transformation of Arabidopsis plants by the floral dip method [34]. Selection of transgenic plants was done according to Szakazits et al. [35] for the pPZP3425 vectors and Ali et al. [36] for the pMAA-Red derivative.

For the overexpression constructs of WRKY33 (*At2g38470*) and MKK4 (At1g51660), the protein coding sequence was amplified from cDNAs using RNA isolated from 14-d-old roots. The primers used to amplify the WRKY33 coding sequence (cWRKY33forNco and cWRKY33revBam) from cDNA are given in Table S1. In case of WRKY33 overexpression constructs, the WRKY coding sequence was driven by three promoters in the expression cassette of the vector pPZP3425 [35]. The CaMV35S promoter was used for constitutive overexpression [37], while Arabidopsis *Pdf2.1* and *MIOX5* promoters were used for driving the expression of WRKY33 specifically in syncytia [13], [38]. In case of MKK4, only the *Pdf2.1* promoter was used for its syncytium specific expression using the vector pMAA-Red [36]. For amplification of the genes from cDNA or genomic DNA, Phusion High-Fidelity DNA Polymerase (Thermo Scientific) was used. All constructs were confirmed by sequencing.

The *Pdf2.1*::pPZP3425 construct has been described (Siddique et al. 2011). The *MIOX5* promoter was amplified from genomic DNA (ecotype Col) using the primers listed in Table S1 which introduced an upstream EcoRI site and an NcoI site at the start codon. After digestion with these enzymes, this PCR fragment was used to replace the CaMV promoter in pPZP3425 digested with the same enzymes resulting in *MIOX5*::pPZP3425.

NcoI and BamHI restriction sites were used for insertion of coding sequences in the vectors. The coding sequence of MKK4 has an endogenous BamHI site which was removed by an overlapping PCR using the primers MKK4forNco2, MKK4Mfor, MKK4Mrev and MKK4revBam (Table S1). These primers also introduced 2 mutations (T224D and S230D) which render MKK4 constitutively active [39], [40], resulting in MKK4^DD^.

Eight homozygous lines from the WRKY33 overexpression construct with CaMV promoter were evaluated for transcript abundance using semi-quantitative reverse transcriptase PCR (RT-PCR) using primers WRKY33RTfor and WRKY33RTrev (Table S1). For RT-PCR RNA was isolated from 15 days old seedlings and two lines showing high expression levels were selected for nematode resistance tests. For the constructs *MIOX5::WRKY33*, *Pdf2.1*::*WRKY33*, and *Pdf2.1::MKK^DD^* selection at the seedling level by RT-PCR was not possible because the promoters are not active in these tissues. Therefore 8 lines each were challenged with *H. schachtii* in preliminary resistance tests to select lines according to their susceptibility (Figure S1). For the *WRKY33* constructs 2 transgenic lines were analyzed in detail while for the *Pdf2.1::MKK^DD^* construct 3 lines were further analyzed.

The promoter region 1700 bp upstream the start codon of the *WRKY17* gene (*At2g24570*) was amplified by PCR (Phusion High-Fidelity DNA Polymerase from Thermo Scientific) using 50 ng Arabidopsis Col genomic DNA as template. The primer pair used for amplification of the promoter region was promWRKY17forEcoRI and prom*WRKY17*revNcoI (Table S1). Primers included restriction sites for EcoRI and NcoI for subsequent cloning into the binary vector pMAA-Red [36]. During the cloning procedure the 35S promoter was exchanged by the promoter fragment of *WRKY17*. Seven homozygous lines were developed for the *pWRKY17*::GUS fusion construct and their expression pattern was tested. Four lines with a similar expression pattern were selected and one of these was used for a detailed GUS expression analysis.

The *WRKY33*::GUS line (Lippok et al. 2007), overexpression line *35S*::*WRKY6-*9, *wrky6-2* mutant, and the *pWRKY6*::GUS line [32] were all provided by Dr. Imre E. Somssich (Max-Planck-Institute for Plant Breeding, Department of Plant-Microbe Interactions, Koln, Germany). The *wrky33-1* mutant line [28] was provided by Dr. Michael K. Deyholos (Department of Biological Sciences, University of Alberta, Edmonton, Canada). The single and double mutants of *WRKY11* and *WRKY17* (*wrky11-1*, *wrky17-1* and *wrky11-wrky17*) and promoter::GUS line of *WRKY11* [41] were provided by Dr. Thomas Kroj (Laboratory of Plant-Microorganism Interactions, Castanet Tolosan, France). The phytoalexin deficient *pad3-1* mutant line (N3805) was obtained from the Nottingham Arabidopsis stock center.

### Nematode infection and resistance test

*H. schachtii* cysts were harvested from sterile *in vitro* stock cultures propagated on mustard (*Sinapsis alba* cv Albatros) roots growing on 0.2x concentrated Knop medium supplemented with 2% sucrose [3]. The cysts were soaked in 3 mM ZnCl_2_ to stimulate hatching of J_2_ larvae under sterile conditions. The J_2_ larvae were then washed three times in sterile water and resuspended in 0.5% (w/v) Gelrite (Duchefa, Haarlem, The Netherlands) before inoculation. Different overexpression and mutant lines together with wild type plants were grown on Knop medium in 9 cm Petri dishes (10 seedlings per plate) as described above in a growth chamber with a 16 h light/8 h dark cycle at 23°C. The roots of twelve-d-old Arabidopsis plants were inoculated under sterile conditions with about 50–60 juveniles per plant. Before inoculation with *H. schachtii*, total root length of each plant was estimated as described [42]. At 14 dpi, 15 syncytia associated with female nematodes were randomly selected and photographed under an inverse microscope (Axiovert 200 M; Zeiss, Hallerbergmoos, Germany) having an integrated camera (AxioCam MRc5; Zeiss). Syncytia and female nematodes were measured using the AxioVision 4 software (Zeiss, Hallerbergmoos, Germany). Examples are shown in Figure S2. Afterwards, female and male nematodes were counted and the number of males and females per cm of root length was calculated at 15 dpi. Three independent biological replicates were performed with a gap of 1 week between the replicates. One replicate consisted of 5 plates with 10 plants per plate; thus a total of 150 plants was analysed per line. The data regarding number of nematodes and sizes of nematodes and syncytia were analysed using single factor ANOVA (P<0.05, P<0.01) and T-Test (P<0.05).

### Histochemical GUS analysis

Histochemical detection of GUS activity was done with X-gluc (Biomol, Hamburg, Germany) as substrate in 0.1 M sodium phosphate buffer pH 7.0 containing 0.1% Triton-X 100, 0.5 mM K_3_[Fe(CN)_6_], 0.5 mM K_4_[Fe(CN)_6_] and 10 mM Na_2_EDTA [43]. For GUS staining of syncytia infection was done as described above and infected roots from different time points were then incubated with X-gluc overnight at 37°C. The stained syncytia and uninfected roots were photographed as described above.

### RNA isolation

Plant samples were immediately frozen in liquid nitrogen and total RNA was isolated using a NucleoSpin RNA Plant kit (Machery & Nagel, genXpress) according to the manufacturer’s instructions and stored immediately at −80°C. The RNA preparation included DNase digestion. However, since this DNase treatment did not completely remove the DNA in the sample, the remaining DNA was digested using Ambion DNA-free DNase Treatment and Removal Reagents (Invitrogen) for some experiments. RNA was quantified using NanoDrop (NanoDrop 2000c from PEQLAB).

### Reverse Transcriptase (RT-PCR) and quantitative Real Time RT-PCR (qRT-PCR)

RT-PCR was done using the RT-PCR Master Mix (USB) following the manufacturer’s instructions. For cDNA synthesis Superscript III reverse transcriptase (Invitrogen) and random primers (oligo(dN)6) according to the manufactures instructions were used. The RNA concentration was approximately 100 ng/µl and 10 µl was used in a total volume of 20 µl. The qRT-PCR was performed on an ABI PRISM 7300 Sequence Detector and results were calculated using the Sequence Detection Software SDS v2.0 (Applied BioSystems). Each qRT-PCR sample contained 12.5 µl Platinum SYBR Green qPCR SuperMix with UDG and ROX (Invitrogen), 2 mM MgCl_2_, 0.5 µl forward and reverse primer (10 µM), 2 µl cDNA and water to make a 25 µl total reaction volume. The primer pairs used for qRT-PCR for *WRKY33* were WRKY33qRTfor and WRKY33qRTrev (Table S1). Dissociation runs were performed to exclude the formation of primer dimers. The *18S* gene was used as an internal reference and relative expression was calculated by the (1+E)^−ΔΔCt^ method [44].

### Statistical analysis of microarray data

Affymetrix CEL files were analyzed using packages of the Bioconductor suite (www.bioconductor.org). Our approach follows established practice and has been described in detail before [4]. In brief, for the statistical tests, individual gene variances have been moderated using an Empirical Bayes approach as in related studies [13]. Further information is available in the online methods (File S1). Tests were restricted to the 59 WRKY genes included on the GeneChip representing most of the 66 genes recognised by Wang et al. [45] and containing the originally described WRKY gene subgroups [16]. This considerably increased the statistical power of the testing procedure as it reduces the necessary correction for massive multiple testing.

## Results

### Expression of *WRKY* genes in syncytia

Arabidopsis contains 66 *WRKY* genes [45] with 59 represented by the ATH1 GeneChip. Of these, 6 were up-regulated in syncytia as compared to control root segments (Table 1). This becomes also visible from the MA plot shown in Figure S3. Among the up-regulated genes was also *WRKY23*, which has been shown to be important for nematode development [21]. A comparison of 5 and 15 dpi syncytia uncovered only 2 genes that were significantly differently expressed; *WRKY40* and *WRKY54* (Figure S3). Both genes were up-regulated in older syncytia and *WRKY54* was also significantly up-regulated in syncytia compared to control root segments. Almost half of the *WRKY* genes (28) were significantly down-regulated in syncytia as compared to control root segments (Table 1 and Figure S4). Down-regulation of the expression of the *WRKY* genes *WRKY6*, *WRKY11*, *WRKY17*, and *WRKY33* indicated that expression of these genes in syncytia would be detrimental to the development of the nematode.

**Table 1:** Expression of WRKY genes in syncytia and control root segments.

ID	Gene	Root	Syn	Syn vs Root Root	q
*At4G31550*	***WRKY11***	5.92	3.62	−2.30	**1.3E-4**
*At2G34830*	***WRKY35***	6.18	2.73	−3.45	**1.3E-4**
*At4G01720*	***WRKY47***	4.83	2.51	−2.32	**1.3E-4**
*At1G69810*	***WRKY36***	6.73	2.49	−4.24	**1.4E-4**
*At2G47260*	*WRKY23*	4.80	6.71	1.91	**2.3E-4**
*At2G40750*	*WRKY54*	4.58	5.84	1.26	**3.2E-4**
*At2G38470*	***WRKY33***	9.18	4.07	−5.11	**3.2E-4**
*At1G29280*	***WRKY65***	8.45	3.97	−4.48	**3.2E-4**
*At1G62300*	***WRKY6***	8.07	4.56	−3.51	**3.7E-4**
*At3G58710*	***WRKY69***	8.62	5.41	−3.20	**0.1%**
*At4G18170*	*WRKY28*	4.71	6.25	1.54	**0.1%**
*At2G24570*	***WRKY17***	6.20	3.57	−2.62	**0.1%**
*At4G22070*	***WRKY31***	2.78	1.92	−0.86	**0.1%**
*At1G68150*	***WRKY9***	6.30	2.13	−4.17	**0.1%**
*At2G23320*	***WRKY15***	6.77	5.32	−1.45	**0.1%**
*At2G30250*	***WRKY25***	4.40	3.15	−1.25	**0.1%**
*At5G13080*	***WRKY75***	6.02	3.10	−2.93	**0.1%**
*At1G30650*	***WRKY14***	4.77	2.10	−2.67	**0.1%**
*At1G18860*	***WRKY61***	3.80	2.17	−1.64	**0.1%**
*At4G30935*	*WRKY32*	5.41	6.46	1.05	**0.2%**
*At4G24240*	***WRKY7***	4.64	3.51	−1.14	**0.2%**
*At2G25000*	***WRKY60***	4.00	3.24	−0.76	**0.2%**
*At5G52830*	***WRKY27***	6.26	3.78	−2.48	**0.3%**
*At5G49520*	*WRKY48*	2.62	4.54	1.92	**0.6%**
*At5G15130*	***WRKY72***	3.53	2.33	−1.20	**0.6%**
*At2g04880*	***WRKY1/ZAP1***	7.27	6.33	−0.95	**0.8%**
*At5g07100*	***WRKY26***	4.42	2.91	−1.51	**1.8%**
*At3g01080*	***WRKY58***	4.35	3.76	−0.59	**1.8%**
*At3g01970*	*WRKY45*	4.55	5.44	0.90	**1.8%**
*At1g64000*	***WRKY56***	2.89	2.39	−0.49	**2.2%**
*At2g46130*	***WRKY43***	3.01	2.29	−0.71	**3.5%**
*At4g23810*	***WRKY53***	5.22	3.80	−1.41	**3.5%**
*At1g13960*	***WRKY4***	5.56	4.72	−0.84	**3.6%**
*At3g04670*	***WRKY39***	6.73	5.86	−0.88	**5.0%**
*At4g04450*	*WRKY42*	3.11	2.72	−0.39	6.6%
*At5g28650*	*WRKY74*	4.93	5.44	0.51	6.7%
*At5g45260*	*WRKY52/RRS1*	5.94	4.66	−1.27	10.0%
*At2g03340*	*WRKY3*	4.21	3.80	−0.42	13.9%
*At3g56400*	*WRKY70*	4.60	4.26	−0.34	16.0%
*At2g30590*	*WRKY21*	3.74	4.13	0.39	17.6%
*At1g80840*	*WRKY40*	6.46	7.09	0.62	19.1%
*At4g26640*	*WRKY20*	6.32	5.91	−0.41	26.8%
*At5g22570*	*WRKY38*	2.30	2.48	0.18	34.3%
*At1g29860*	*WRKY71*	3.33	3.19	−0.14	45.1%
*At4g26440*	*WRKY34*	2.32	2.22	−0.11	50.8%
*At2g44745*	*WRKY12*	2.90	2.98	0.08	73.4%
*At1g55600*	*WRKY10*	2.98	2.91	−0.07	80.2%
*At4g31800*	*WRKY18*	5.23	5.08	−0.14	80.3%
*At5g24110*	*WRKY30*	2.78	2.82	0.04	87.7%
*At2g40740*	*WRKY55*	3.70	3.65	−0.05	87.7%
*At4g01250*	*WRKY22*	3.84	3.91	0.08	87.8%
*At2g46400*	*WRKY46*	4.13	4.17	0.05	87.8%
*At2g37260*	*WRKY44/TTG2*	2.62	2.58	−0.03	87.8%
*At5g56270*	*WRKY2*	4.69	4.72	0.03	87.8%
*At1g66550*	*WRKY67*	2.25	2.28	0.03	91.7%
*At1g69310*	*WRKY57*	3.06	3.08	0.02	92.8%
*At4g39410*	*WRKY13*	3.60	3.57	−0.03	92.8%
*At5g46350*	*WRKY8*	2.83	2.84	0.01	94.7%
*At1g80590*	*WRKY66*	2.64	2.64	−0.01	96.4%

The data for microaspirated syncytia at 5 dpi and 15 dpi were compared with control root segments (containing elongation zone without root tip). Columns 3 and 4 contain normalized expression values on a log_2_ scale. Column 5 shows the differences (fold changes) between the pairwise samples as normalized log_2_ ratios (see the Online Methods section for details). The q-values in column 6 indicate significance after correction for multiple testing controlling the False Discovery Rate. Genes with a significant up- or down-regulation (q<5%) are marked in bold in column 6. Genes with significant down-regulation are in addition shown with the gene symbol in bold.

### *WRKY6, WRKY11, WRKY17*

**Figure 1:**
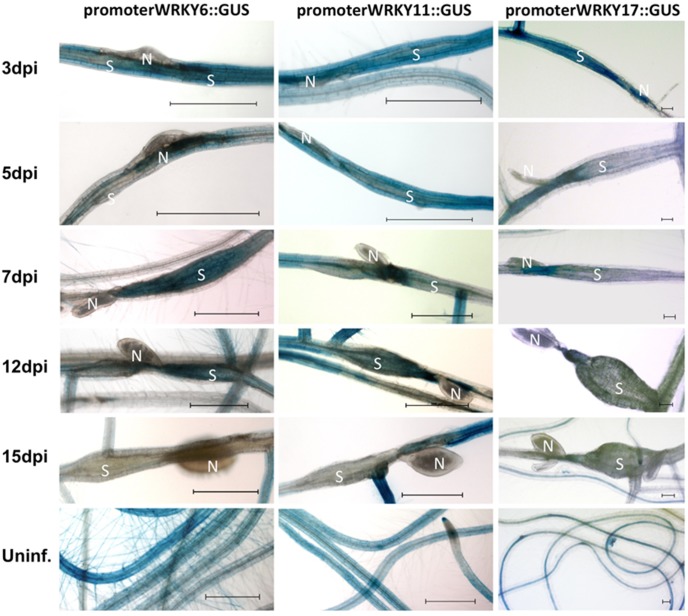
GUS expression of *WRKY6*, *WRKY11* and *WRKY17* in syncytia. GUS staining of promoter::GUS lines for *WRKY6*, *WRKY11* and *WRKY17* was performed for 3, 5, 7, 12, and 15 dpi syncytia and uninfected roots. N = nematode, S = syncytia and bar = 500 µm in case of *WRKY6* and *WRKY11* and bar = 100 µm in case of *WRKY17*.

We tested promoter::GUS lines for *WRKY6*, *WRKY11*, and *WRKY17*. These GUS lines showed GUS staining of uninfected roots and of younger syncytia but no staining in older syncytia (Figure 1). For *WRKY6* and *WRKY11*, the down-regulation was visible within the syncytium starting at 3 dpi but there was still GUS staining visible up to 12 dpi in the tissues surrounding the syncytium. At 15 dpi also the tissues surrounding syncytia did not show any GUS staining. For *WRKY17* the down-regulation of GUS expression occured quickly with GUS staining at 5 dpi only at the end of the syncytium where the nematode inserted its stylet. Thus, for *WRKY6*, *WRKY11*, and *WRKY17* the GUS lines confirmed the down-regulation in the syncytia that was found by GeneChip analysis of syncytia (Table 1).

**Figure 2:**
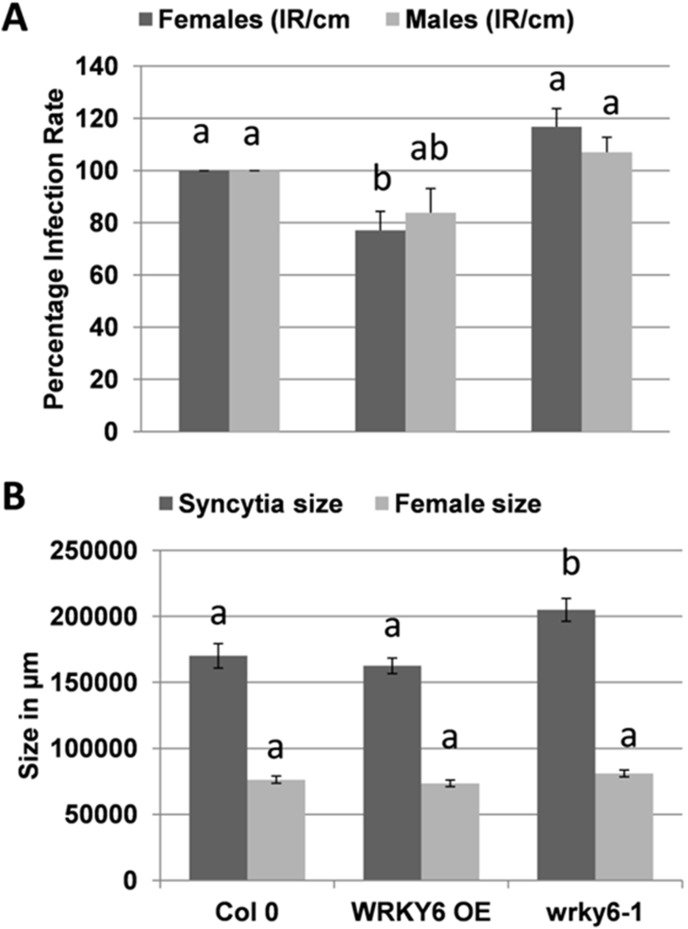
Nematode resistance test for *WRKY6* overexpression line and mutant. The resistance of overexpression line of *WRKY6* along with its knock out mutant compared to wild type plants after infection with *H. schachtii*. A: Number of male and female nemaodes per cm of root length calculated at 15 dpi setting the wild type as 100%. Different letters indicate significant differences (P<0.05; ANOVA and LSD). The statistical significance was determined by three independent replicates. Values are means ± SE, n = 15. The bar shows standard error for the mean. B: Size of female syncytia and female nematodes at 14 dpi. Ten syncytia were selected randomly from three independent replicates (total = 30) and the size of syncytia and associated female nematodes was determined. Data were analysed for significance difference using ANOVA (P<0.05) and LSD. Values are means ± SE.

Increasing the expression of these genes in syncytia might therefore reduce the susceptibility of the plant. For *WRKY6*, we tested the overexpression line and mutant published by Somssich and colleagues [32]. The infection assay with these lines gave mixed results. The number of females was significantly reduced on the overexpression line. The number of males was also reduced on these lines but the difference was not significant. On the mutant there was a tendency to higher numbers of females and males as compared to the wild type but again these differences were not significant (Figure 2A). Syncytium and female size were also not different from the wild type on the overexpression line. The syncytia induced on the *wrky6* mutant on the other hand were significantly larger than those on wild type plants but the size of females did not differ compared to those developing on wild type plants (Figure 2B).

**Figure 3:**
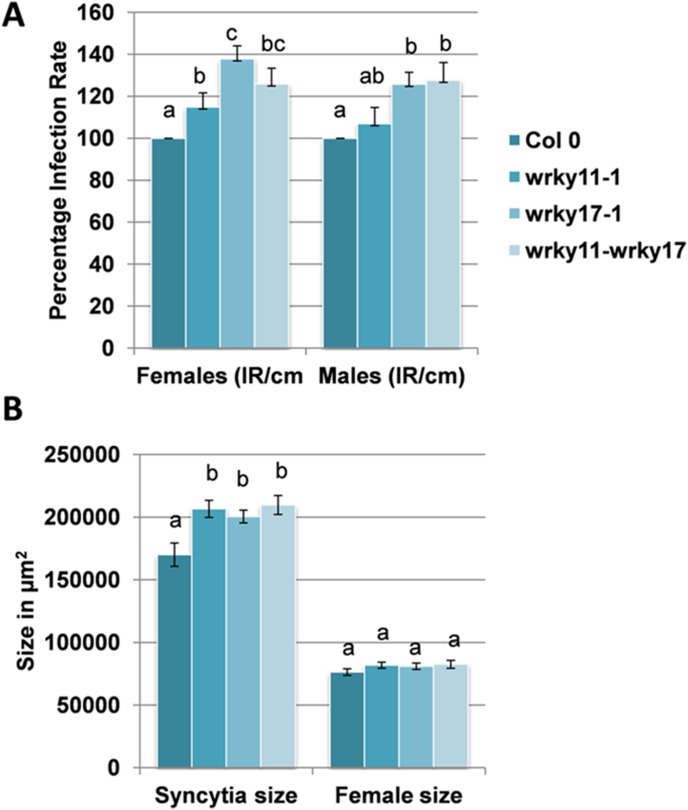
Nematode resistance test for *WRKY11* and *WRKY17* mutants. The resistance of single and double mutants of *WRKY11* and *WRKY17* as compared to wild type plants after infection with *H. schachtii*. A: Number of male and female nemaodes per cm of root length calculated at 15 dpi setting the wild type as 100%. Different letters indicate significant differences (P<0.05; ANOVA and LSD). The statistical significance was determined by three independent replicates. Values are means ± SE, n = 15. The bar shows standard error for the mean. B: Size of female syncytia and female nematodes at 14 dpi. Ten syncytia were selected randomly from three independent replicates (total = 30) and the size of syncytia and associated female nematodes was determined. Data were analysed for significance difference using ANOVA (P<0.05) and LSD. Values are means ± SE.

For *WRKY11* and *WRKY17* the single mutants and a double mutant [41] were tested. The *wrky17* mutant was more susceptible and supported approximately 40% more females and 20% more males than the wild type plants (Figure 3A). The effect of the *wrky11* mutant was not as strong and the number of females was significantly increased by only 20% while the difference for male nematodes was not significant. The double mutant was not significantly different from the *wrky17* mutant. The size of syncytia was increased on all mutants and again the double mutant was not different than the *wrky17* mutant and also the *wrky11* mutant (Figure 3B). No effect was found on the size of female nematodes on all mutants (Figure 3B).

### *WRKY33*

**Figure 4:**
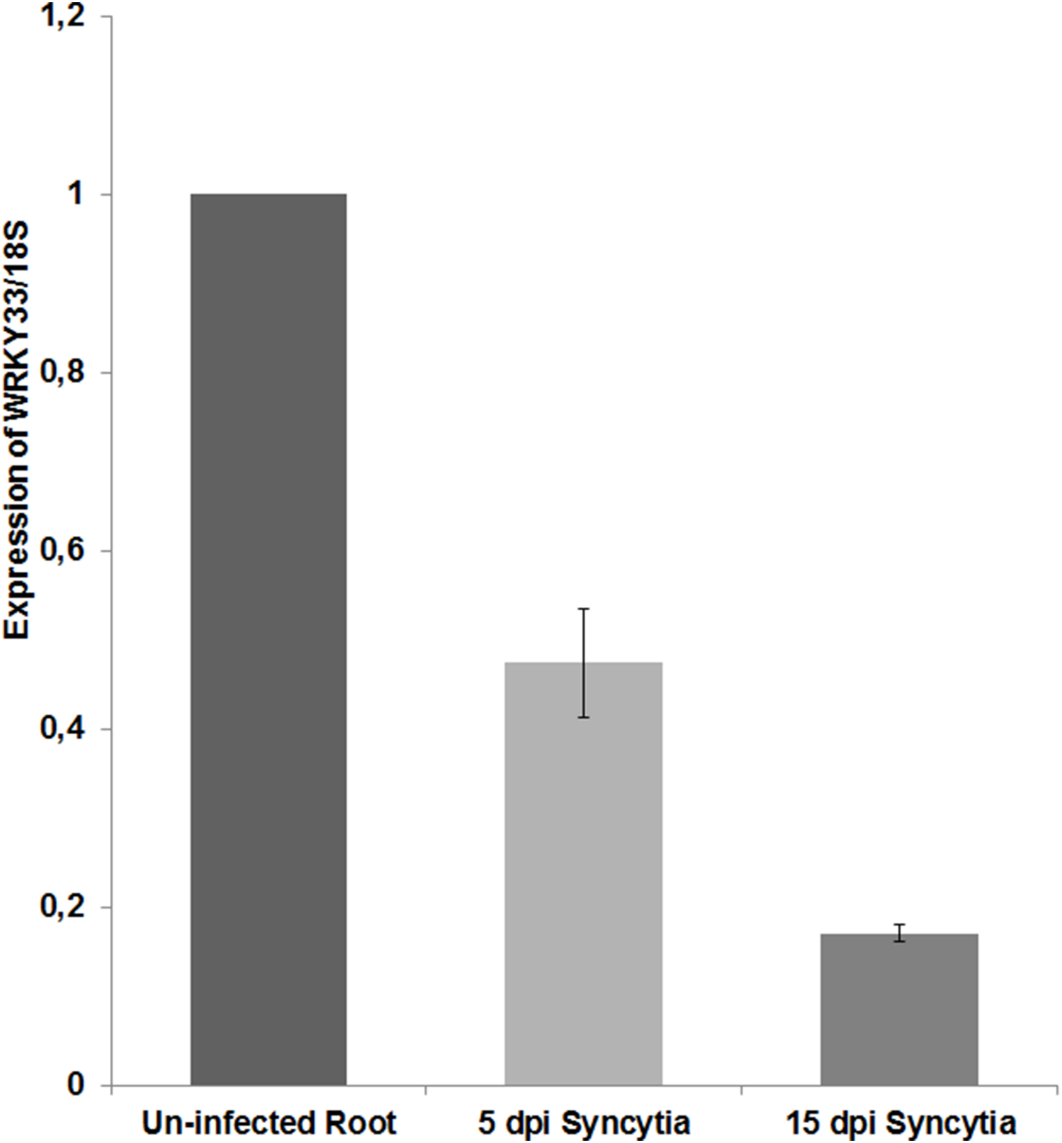
Expression of *WRKY33* in response to nematode infection. Expression of *WRKY33* in wild type plants was determined by qRT-PCR in 5 and 15 dpi syncytia and uninfected root segments (containing elongation zones without root tips from 15-d-old seedling). The data included three independent biological and three technical replicates. The *WRKY33* expression values are relative to un-infected control roots and were normalized using *18S* as a housekeeping gene. Values are means ± SE, n = 3. The bar shows standard error for the mean.

**Figure 5:**
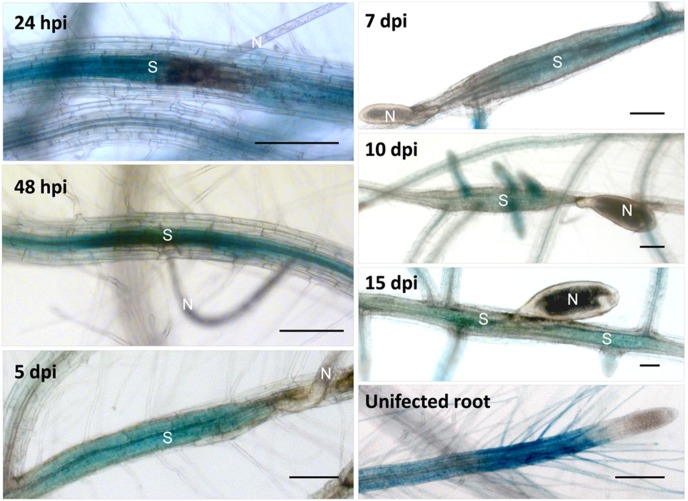
Analysis of *pWRKY33*::GUS expression in syncytia. GUS staining of a *pWRKY33*::GUS line was performed for 1, 2, 5, 7, 10 and 15 dpi syncytia and uninfected roots. N = nematode, S = syncytia and bar = 100 µm.

The *WRKY33* gene was among the most strongly down-regulated genes in syncytia and was therefore studied in more detail. We used qRT-PCR on syncytia cut from infected roots to confirm the GeneChip data. At 5 dpi expression of *WRKY33* was down-regulated by 50% and at 15 dpi by 80% (Figure 4). This expression was also confirmed using a promoter::GUS line (Figure 5). In uninfected seedlings, *WRKY33* was expressed in roots, especially in the root elongation zone, and in root hairs but not in root tips. In syncytia expression became gradually down-regulated. Syncytia at 24 and 48 hpi (hours post inoculation) still showed GUS expression while GUS staining was weaker at 5 and 7 dpi and almost undetectable at 10 and 15 dpi. Together, all these results showed that expression of *WRKY33* is down-regulated during the development of syncytia.

**Figure 6:**
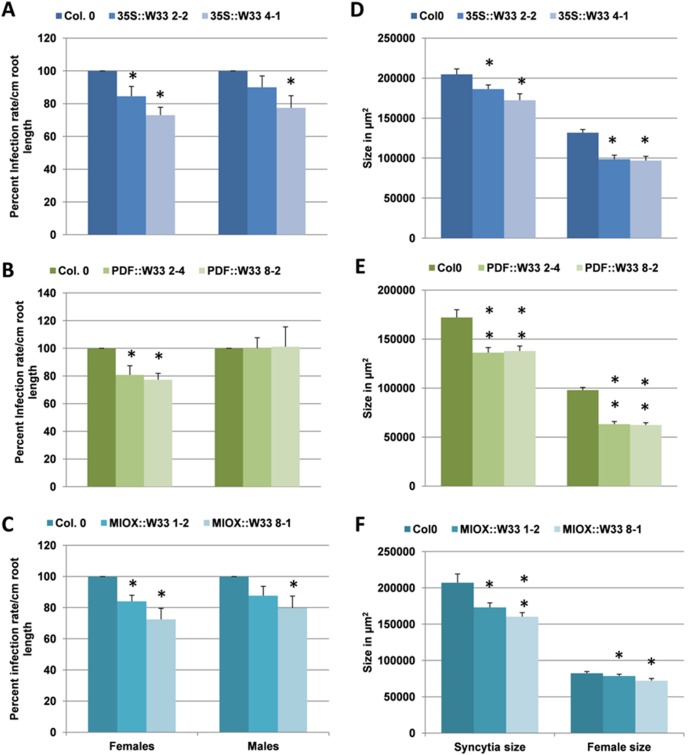
Nematode resistance test for *WRKY33* overexpression lines. The resistance of overexpression lines of *WRKY33* with constitutive 35S promoter (A&D) and syncytia specific promoters *Pdf2.1 (B&E) and Miox5 (C&F)* was compared to wild type plants after infection with *H. schachtii*. A–C: Number of male and female nemaodes per cm of root length calculated at 15 dpi setting the wild type as 100%. Asterisks indicate significant differences (*, P<0.05 and **, P<0.01; ANOVA and LSD). The statistical significance was determined by three independent replicates. Values are means ± SE, n = 15. The bar shows standard error for the mean. D–F: Size of female syncytia and female nematodes at 14 dpi. Ten syncytia were selected randomly from three independent replicates (total = 30) and the size of syncytia and associated female nematodes was determined. Data were analysed for significance difference using ANOVA (P<0.05) and LSD. Values are means ± SE.

**Figure 7:**
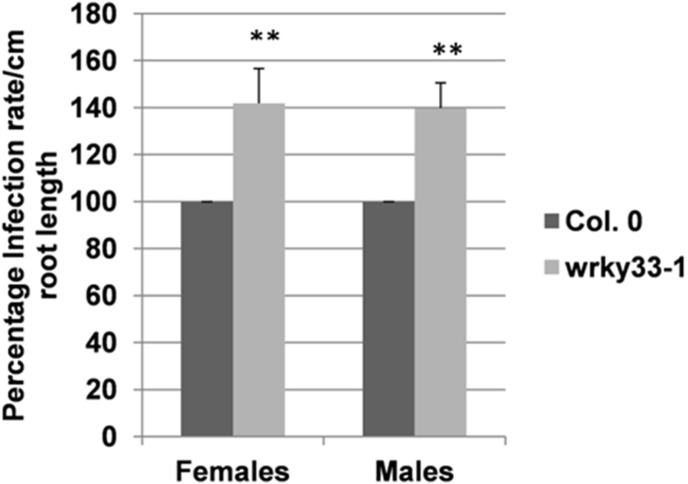
Nematode resistance test for *wrky33* knock out mutant. The resistance of *wrky33* knock out mutant (*wrky33-1*) was compared to wild type plants after infection with *H. schachtii*. A: Number of male and female nemaodes per cm of root length calculated at 15 dpi setting the wild type as 100%. Asterisks indicate significant differences (P<0.01; T-test). The statistical significance was determined by three independent replicates. Values are means ± SE, n = 15. The bar shows standard error for the mean.

In order to study the effect of WRKY33 on nematode development we produced Arabidopsis lines expressing WRKY33 driven either by the CaMV 35S promoter, the *Pdf2.1* [38], or the *MIOX5* [13] promoter as described in Materials and Methods. For each construct we selected two lines and tested them in a nematode infection assay. All lines showed a 20–30% reduction in the number of females as compared to the wild type plants (Figure 6, A–C) while the number of males was not significantly reduced in all lines. All lines also affected the size of syncytia and the size of the females which was significantly reduced in all lines (Figure 6, D–F). Conversely, the *wrky33* mutant [28] was more susceptible than the wild type and supported approximately 40% more females and males as compared to the wild type (Figure 7).

The regulation and function of WRKY33 has been intensively studied. WRKY33 is phosphorylated by MPK3/MPK6, leading to activation and further to induction of ethylene biosynthesis [46] and camalexin biosynthesis [26]. MPK3/MPK6 itself are targets of MKK4 and MKK5 [47]. We therefore tested if the expression of a constitutive active MKK4 (MKK4^DD^) might also lead to a similar effect as the overexpression of WRKY33. Since the MAP kinase pathway that activates MPK3/MPK6 is involved in various pathways, a constitutive activation of this pathway might be detrimental to the plant and we used the *Pdf2.1* promoter to drive MKK4^DD^. This promoter is strongly expressed in seeds and syncytia [38].

**Figure 8:**
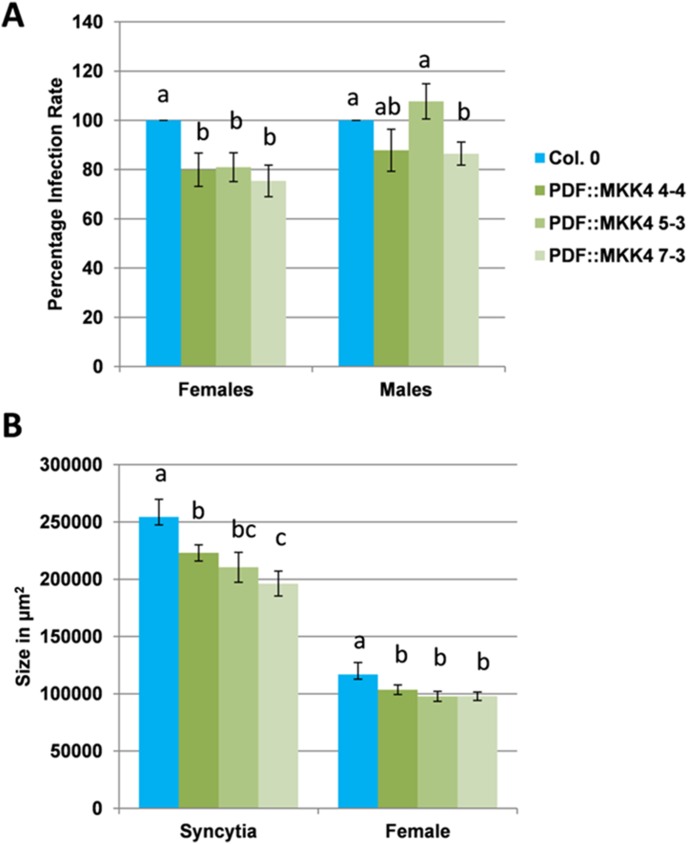
Nematode resistance test for *MKK4^DD^* overexpression lines. The resistance of *MKK4^DD^* lines was compared to wild type plants after infection with *H. schachtii*. A: Number of male and female nemaodes per cm of root length calculated at 15 dpi setting the wild type as 100%. Different letters indicate significant differences (P<0.05; ANOVA and LSD). The statistical significance was determined by three independent replicates. Values are means ± SE, n = 15. The bar shows standard error for the mean. B: Size of female syncytia and female nematodes at 14 dpi. Ten syncytia were selected randomly from three independent replicates (total = 30) and the size of syncytia and associated female nematodes was determined. Data were analysed for significance difference using ANOVA (P<0.05) and LSD. Values are means ± SE.

The *Pdf2.1*::MKK4^DD^ construct was produced as described in Materials and Methods. Three independent homozygous MKK4^DD^ lines were tested for their resistance against *H. schachtii*. All three lines were less susceptible as shown by a lower number of female nematodes and a lower number of male nematodes in 2 lines (Figure 8A). Furthermore, all three MKK4^DD^ lines supported smaller syncytia and the females developing on these lines were also smaller than those on wild type plants (Figure 8B).

**Figure 9:**
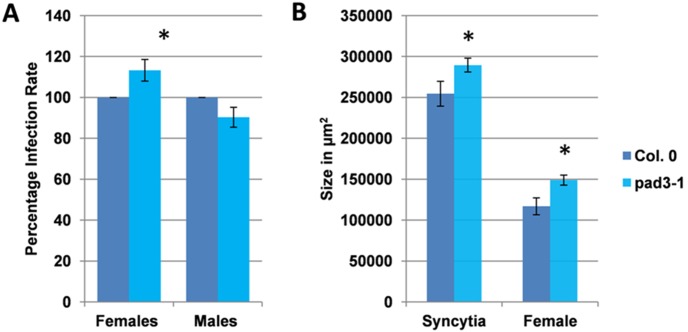
The *pad3* mutant is more susceptible. The resistance of *pad3* knock out mutant compared to wild type plants after infection with *H. schachtii*. A: Number of male and female nemaodes per cm of root length calculated at 15 dpi setting the wild type as 100%. Asterisks indicate significant differences (P<0.05; T-test). The statistical significance was determined by three independent replicates. Values are means ± SE, n = 15. The bar shows standard error for the mean. B: Size of female syncytia and female nematodes at 14 dpi. Ten syncytia were selected randomly from three independent replicates (total = 30) and the size of syncytia and associated female nematodes was determined. Data were analysed for significance difference using T-test. Values are means ± SE.

WRKY33 has been shown to bind to the *PAD3* promoter, thereby inducing expression of the gene coding for CYP71B15, an enzyme which is involved in the last step of camalexin biosynthesis [48], [49]. While we found that WRKY33 overexpression resulted in lower susceptibility to *H. schachtii*, the *wrky33* mutant had the opposite effect. To test if these results might be due to camalexin, the *pad3* mutant which is blocked in camalexin biosynthesis was used. This mutant was more susceptible to *H. schachtii*, with a significantly higher number of females while the number of males was not significantly different from the wild type (Figure 9A). In addition, the *pad3* mutant supported bigger syncytia and cysts (Figure 9B). These results indicate that the enhanced resistance of WRKY33 and MKK4^DD^ overexpressing lines might act at least in part through camalexin, but further work will be needed to clarify as to what extent camalexin is involved.

## Discussion

### Expression of *WRKY* genes in syncytia

WRKY transcription factors are widespread in plants and are involved in many pathways that are important for resistance against abiotic or biotic stresses. We have studied here the role of WRKY transcription factors in Arabidopsis, especially WRKY33, in the interaction with the beet cyst nematode *H. schachtii*. Our analysis revealed a down-regulation of 28 *WRKY* genes in syncytia as compared to control root segments (Table 1). *WRKY33* and others are known to be involved in resistance pathways and such pathways are down-regulated in syncytia [4]. Among the 6 genes that were significantly up-regulated in syncytia according to this study (Table 1) was *WRKY23* which is important for development of *H. schachtii* [21]. The other up-regulated *WRKY* genes included *WRKY54,* which, together with *WRKY70*, has been shown to be a negative regulator of leaf senescence [50] and *WRKY28* which has been implicated in resistance against drought stress and oxidative stress [51]. *WRKY48* is a repressor of basal defense in Arabidopsis and thus its up-regulation would be in favour of nematode development [52]. For the 2 other up-regulated *WRKY* genes, *WRKY45* and *WRKY32*, no specific function has been reported.

The expression of only two *WRKY* genes was significantly different between 5 dpi and 15 dpi syncytia. *WRKY40* and *WRKY54* were both up-regulated in 15 dpi syncytia. *WRKY54* has already been mentioned. *WRKY40*, together with *WRKY18*, has been reported as positive regulator in effector-triggered immunity against *P. syringae* DC3000 expressing the effector AvrRPS4 [53]. On the other hand, a *wrky18 wrky40* double mutant was resistant to the powdery mildew fungus *Golovinomyces orontii* which is otherwise able to infect wild type plants. The resistance was accompanied by accumulation of the phytoalexin camalexin. Thus, these *WRKY* genes are susceptibility factors for *G. orontii*. Powdery mildew fungi are biotrophic pathogens and it would be interesting to test the *wrky18 wrky40* double mutant for resistance against *H. schachtii*.

### *WRKY6*

For *WRKY6* we found a significant decrease of the number of females on the overexpression line and a significant increase in the size of syncytia on the mutant while the other parameters that we measured on these lines were not significantly different from the wild type. *WRKY6* has been reported to be involved in response to boron deficiency [54], phosphate deficiency [55] and senescence [30]. It is also involved in biotic stress responses in Arabidopsis [30]. Data from Yu et al. [31] and Robatzek and Somssich [32] outlined in the introduction support a model where *WRKY6* induces *NPR1* which in turn would lead to the induction of *PR1* and other defense-related genes. Down-regulation of WRKY6 in syncytia might therefore be involved in repressing defense responses of the plant to nematode infection. Such a model is supported by the lower expression level of *NPR1* in syncytia as compared to control root segments [4]. In this regard it is also interesting that expression of AtNPR1 in soybean transgenic roots led to partial resistance against *H. glycines* [56].

### *WRKY11* and *WRKY17*

*WRKY11* and *WRKY17* have been reported as negative regulators of basal resistance [41] because the *wrky11* and *wrky17* mutants were more resistant to *P. syringae*. At the gene expression level, the *wrky11 wrky17* double mutant showed an increased expression of the SA marker gene *PR1* after inoculation with *P. syringae*. On the other hand, in the *wrky11* and the *wrky17* mutants, the expression of the *AOS* and of the *LOX2* gene, both of which are involved in JA biosynthesis, was reduced after inoculation with *P. syringae*. This would lead to a lower JA level after *P. syringae* infection in these mutants. *P. syringae* produces coronatine, a functional homolog of the active form of JA, to induce the JA pathway and thereby block the SA pathway and suppress plant defense reactions against these bacteria [57]. Concerning the interaction with *H. schachtii*, we found the opposite effect, that both mutants were more susceptible, with the *wrky17* mutant being more susceptible than the *wrky11* mutant. In the interaction with *P. syringae* the resistance was more pronounced in the *wrky11* mutant [41]. The reason for this different effect of these mutants is currently unknown. It could be that the different organs that are infected by these pathogens play a role but might also be related to JA production. While *P. syringae* induces the JA-pathway to repress SA-dependent resistance reactions, JA has been shown to induce defense responses against plant pathogenic nematodes. In rice, JA was important for systemic resistance against the root knot nematode *M. graminicola* (Nahar et al. 2013) but in tomato it was reported that an intact JA-pathway was required for susceptibility to *M. incognita* (Bhattarai et al. 2008). In syncytia induced by *H. glycines* in soybean roots the JA biosynthetic pathway is downregulated [58]. With the exception of 12-oxophytodienoate reductase 3 (OPR3), genes for lipoxygenase (LOX1), allene oxide cyclase (AOC), allene oxide synthase (AOS), and OPR1 were down-regulated. This down-regulation was extended to JA-responsive genes such as one coding for a vegetative storage protein. We have recently shown that overexpression of the transcription factor RAP2.6 leads to enhanced resistance against *H. schachtii* in Arabidopsis roots [15]. This resistance was accompanied by an elevated expression of JA-responsive genes during early time points after infection. Thus, downregulation of JA production as through downregulation of *WRKY11* and *WRKY17* might favour nematode development and these WRKY factors therefore are positive regulators of resistance against *H. schachtii*. However, it is currently unknown which JA-induced proteins might lead to a resistance against nematodes.

### *WRKY33*

**Figure 10:**
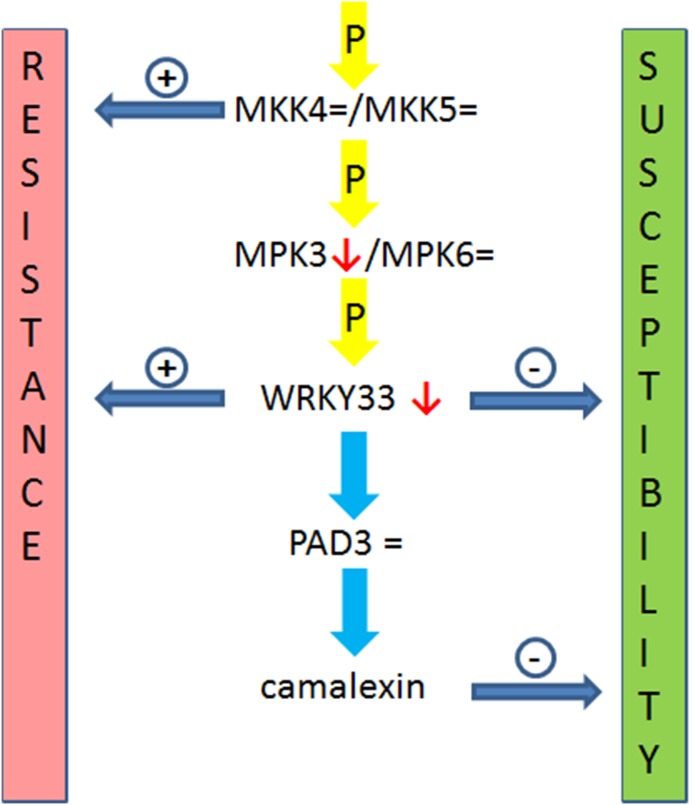
The WRKY33 pathway leading to camalexin as related to *H. schachtii* infection. The phosphorylation cascade involving WRKY33 leading to camalexin. P, phosphorylation. ↓ indicates that the expression of the gene is downregulated in syncytia induced by *H. schachtii* as compared to control root segments while = indicates no change in expression according to data from Szakasits et al. (2009). + and – indicate that increase or decrease leads to enhanced resistance or susceptibility, respectively.

*WRKY33* has been intensively studied by several scientists and is an important regulator of resistance against *B. cinerea* [27], [59]. MKK4 and MKK5 phosphorylate MPK3/MPK6 which in turn phosphorylate WKRY33 [47]. The phosphorylated WRKY33 enters the nucleus to induce the expression of defense-related genes such as those involved in camalexin production [22]. A diagram of this cascade and how it might relate to the interaction with *H. schachtii* is shown in Figure 10. Thus, the Arabidopsis phytoalexin camalexin [26] might be detrimental to nematode development. We found that overexpression of *WRKY33* with different promoters resulted in enhanced resistance against *H. schachtii* while the *wrky33* mutant was more susceptible. Since WRKY33 has to be phosphorylated for activity, a functional phosphorylation cascade is also necessary. In syncytia, *Mpk3* is strongly down-regulated according to the transcriptome analysis of Szakasits et al. [4]. Therefore we tested if a similar effect as through the overexpression of WRKY33 could also be achieved by providing a constitutive active MKK4 which would start the phosphorylation cascade to activate WRKY33. Indeed, also the constitutive active MKK4^DD^ was able to provide enhanced resistance against *H. schachtii*.

Our results indicated that downregulation of *WRKY33* might be important to avoid the production of the phytoalexin camalexin in syncytia. The final step in camalexin biosynthesis is conducted by the P450 enzyme CYP71B15/PAD3 [48] and the *pad3* mutant was indeed more susceptible to *H. schachtii*. However, WRKY33 has also been shown to induce the expression of other genes, for instance those encoding enzymes for ethylene synthesis [46], and further proof is needed for the role of camalexin. If the enhanced resistance in the WRKY33 overexpression lines is in part or solely due to camalexin production could for instance be tested by blocking camalexin production in these lines. In addition, it would be interesting to produce plants with a high constitutive level of camalexin in the roots to test if these would be more resistant to *H. schachtii*. There are a few reports which indicate that phytoalexins may be involved in plant resistance against nematodes as phytoalexins were elicited by nematode infection in resistant lines of several plant species [60], [61].

## Conclusion

WRKY transcription factors are transcriptional regulators of various pathways related to abiotic and biotic stress. We provide evidence that the beet cyst nematode *H. schachtii* manipulates the expression of a large number of these factors to favour its development. Many *WRKY* genes are down-regulated in syncytia and this down-regulation seems to result in the repression of resistance reactions of the plant.

## Supporting Information

Figure S1 **Preliminary resistance tests of overexpression lines with syncytium-specific promoters.**
(PDF)

Figure S2 **Examples of syncytia and attached females for different lines.**
(PDF)

Figure S3 **MA plot (15 dpi syncytium vs. 5 dpi syncytium) for** ***WRKY*** **genes.**
(PDF)

Figure S4 **MA plot (syncytium vs. root) for** ***WRKY*** **genes.**
(PDF)

Table S1 **Primers used in this work.**
(PDF)

File S1 **Online methods bioinformatic analysis.**
(PDF)
