# Supplementary material for: The Beet Cyst Nematode Heterodera schachtii Modulates the Expression of WRKY Transcription Factors in Syncytia to Favour Its Development in Arabidopsis Roots
Source: PLoS One. 2014 Jul 17;9(7):e102360. doi: 10.1371/journal.pone.0102360 (PMC4102525; doi:10.1371/journal.pone.0102360)
Supplement: Figure S1 — Preliminary resistance tests of overexpression lines with syncytium-specific promoters. (PDF) [file pone.0102360.s001.pdf]

Figure S1 – Preliminary resistance tests of overexpression lines with syncytium-specific promoters.

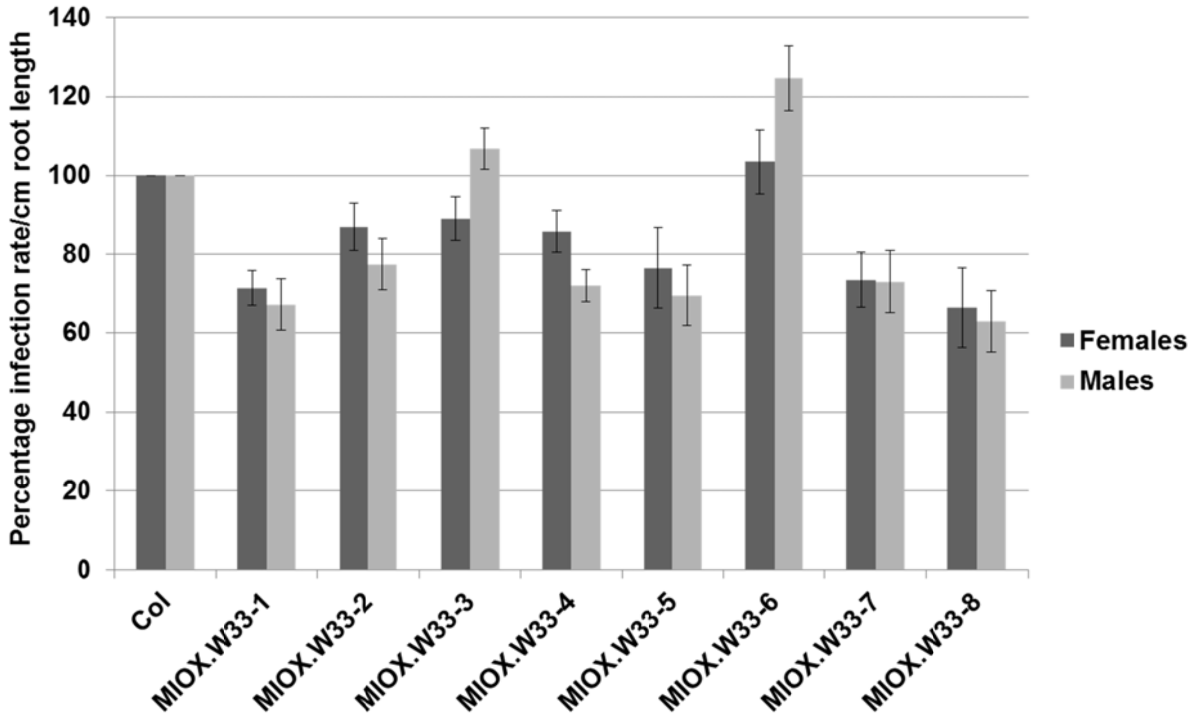

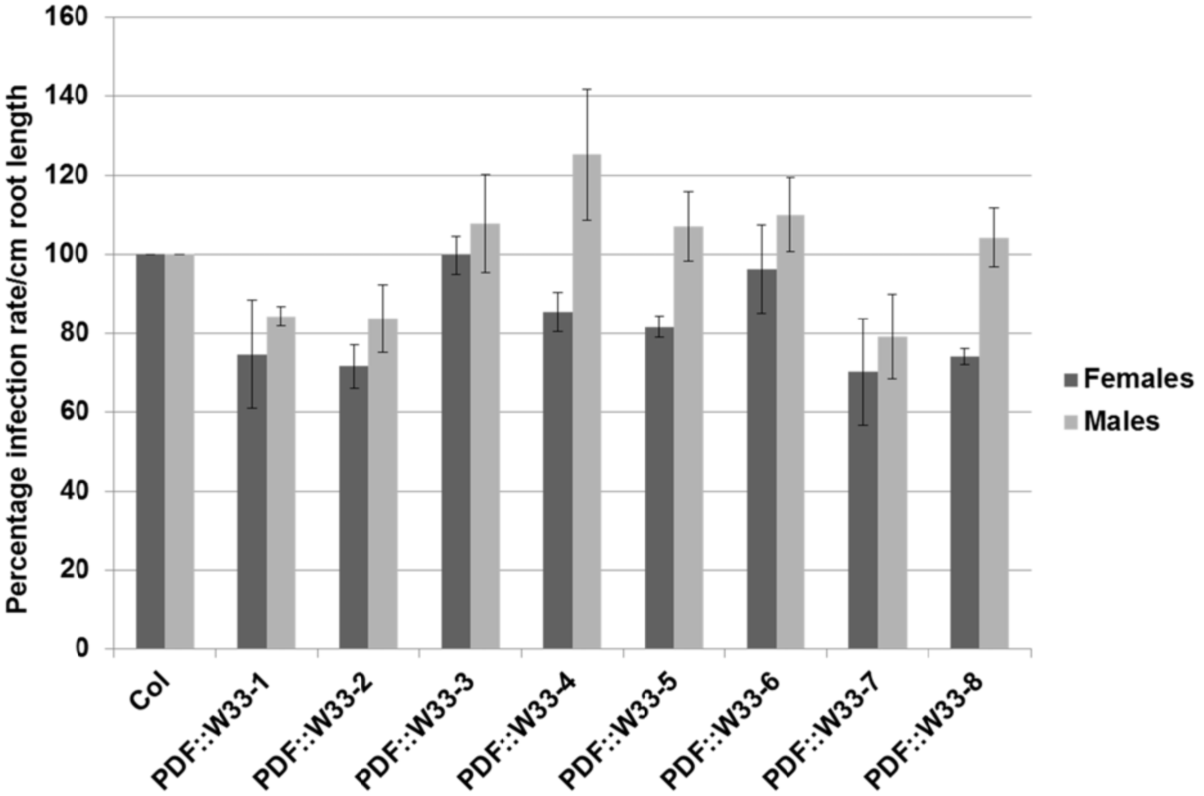

Percentage infection rate/cm root length
160
140
120
100
80
60
40
20
0
Col
PDF::W33-1
PDF::W33-2
PDF::W33-3
PDF::W33-4
PDF::W33-5
PDF::W33-6
PDF::W33-7
PDF::W33-8
Females
Males

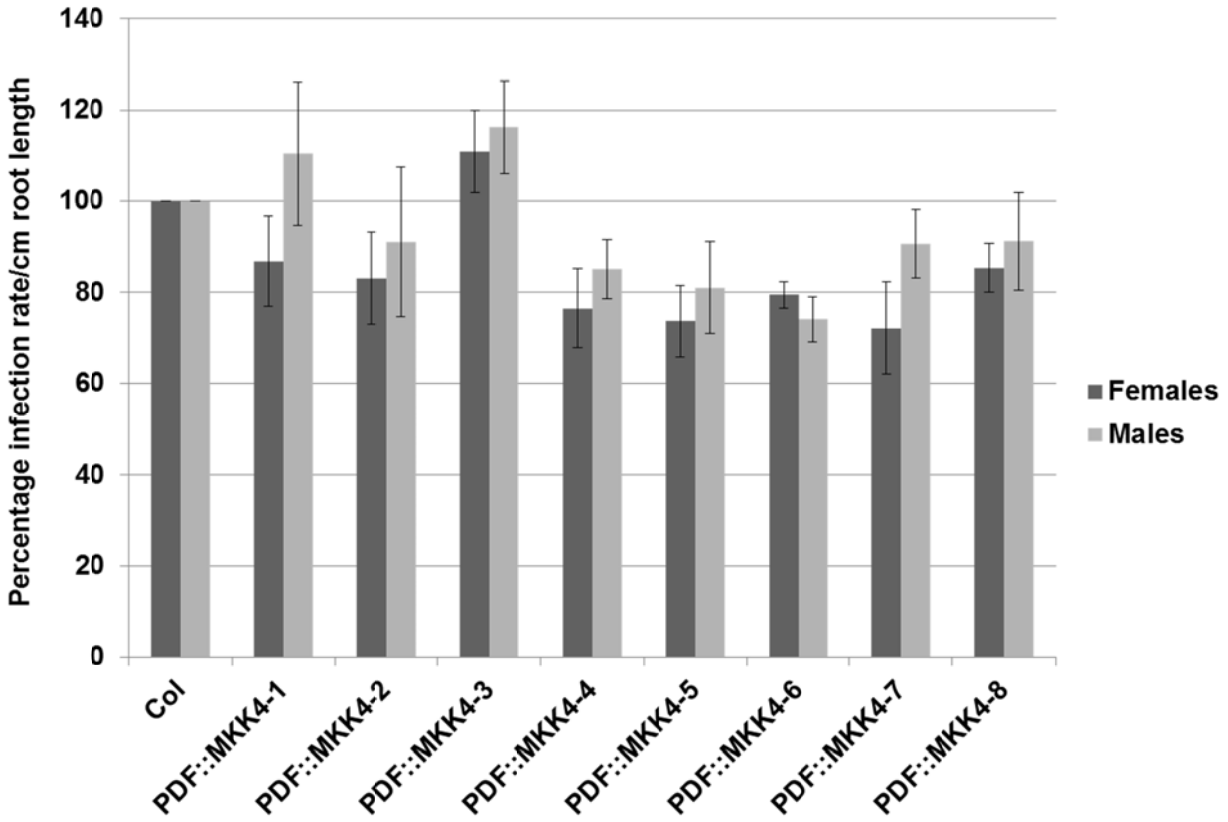
Percentage infection rate/cm root length
0
20
40
60
80
100
120
140
Col
PDF::MKK4-1
PDF::MKK4-2
PDF::MKK4-3
PDF::MKK4-4
PDF::MKK4-5
PDF::MKK4-6
PDF::MKK4-7
PDF::MKK4-8
Females
Males
