# Supplementary material for: The Beet Cyst Nematode Heterodera schachtii Modulates the Expression of WRKY Transcription Factors in Syncytia to Favour Its Development in Arabidopsis Roots
Source: PLoS One. 2014 Jul 17;9(7):e102360. doi: 10.1371/journal.pone.0102360 (PMC4102525; doi:10.1371/journal.pone.0102360)
Supplement: Figure S2 — Examples of syncytia and attached females for different lines. (PDF) [file pone.0102360.s002.pdf]

Figure S2 – Examples of syncytia and attached females for different lines.

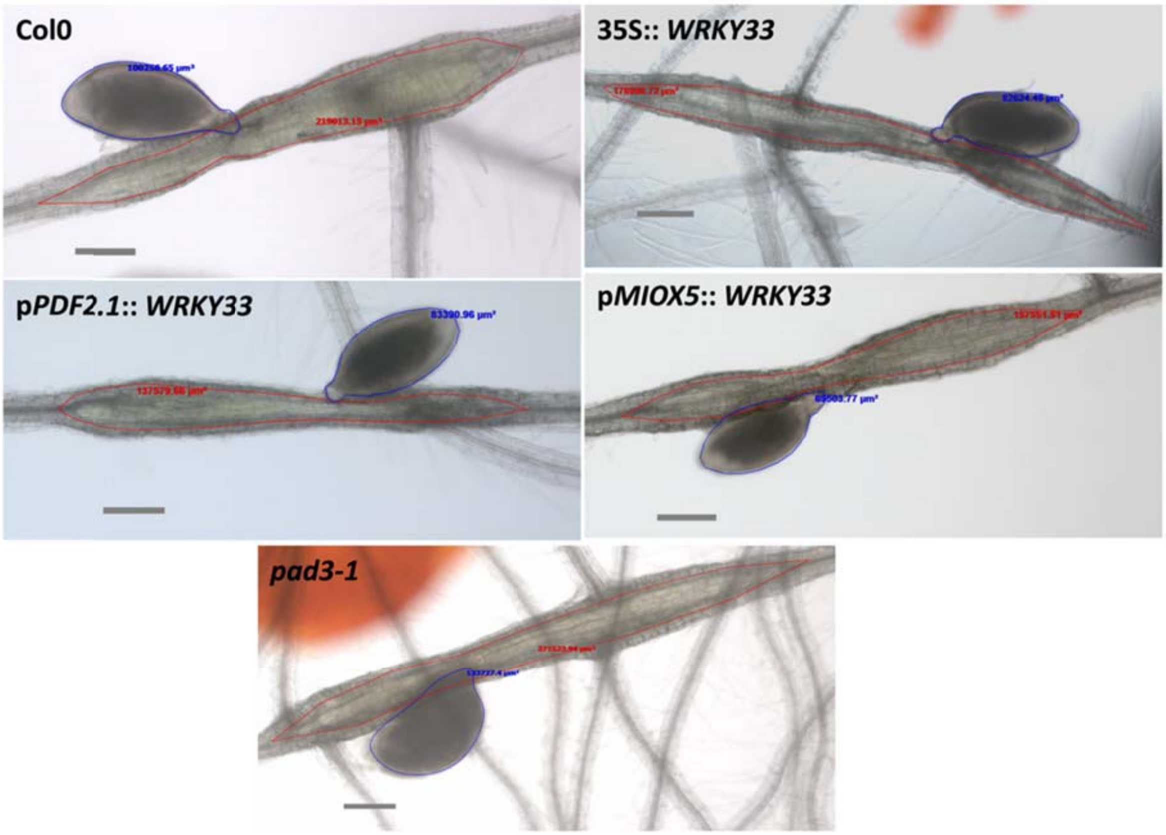

At 14 dpi pictures were taken to measure the size of syncytia and female nematodes.

Bar = 100 µm.
