# Supplementary figures and images for: The Beet Cyst Nematode Heterodera schachtii Modulates the Expression of WRKY Transcription Factors in Syncytia to Favour Its Development in Arabidopsis Roots
Source: PLoS One. 2014 Jul 17;9(7):e102360. doi: 10.1371/journal.pone.0102360 (PMC4102525; doi:10.1371/journal.pone.0102360)

Contrast: Syn15 vsSyn5

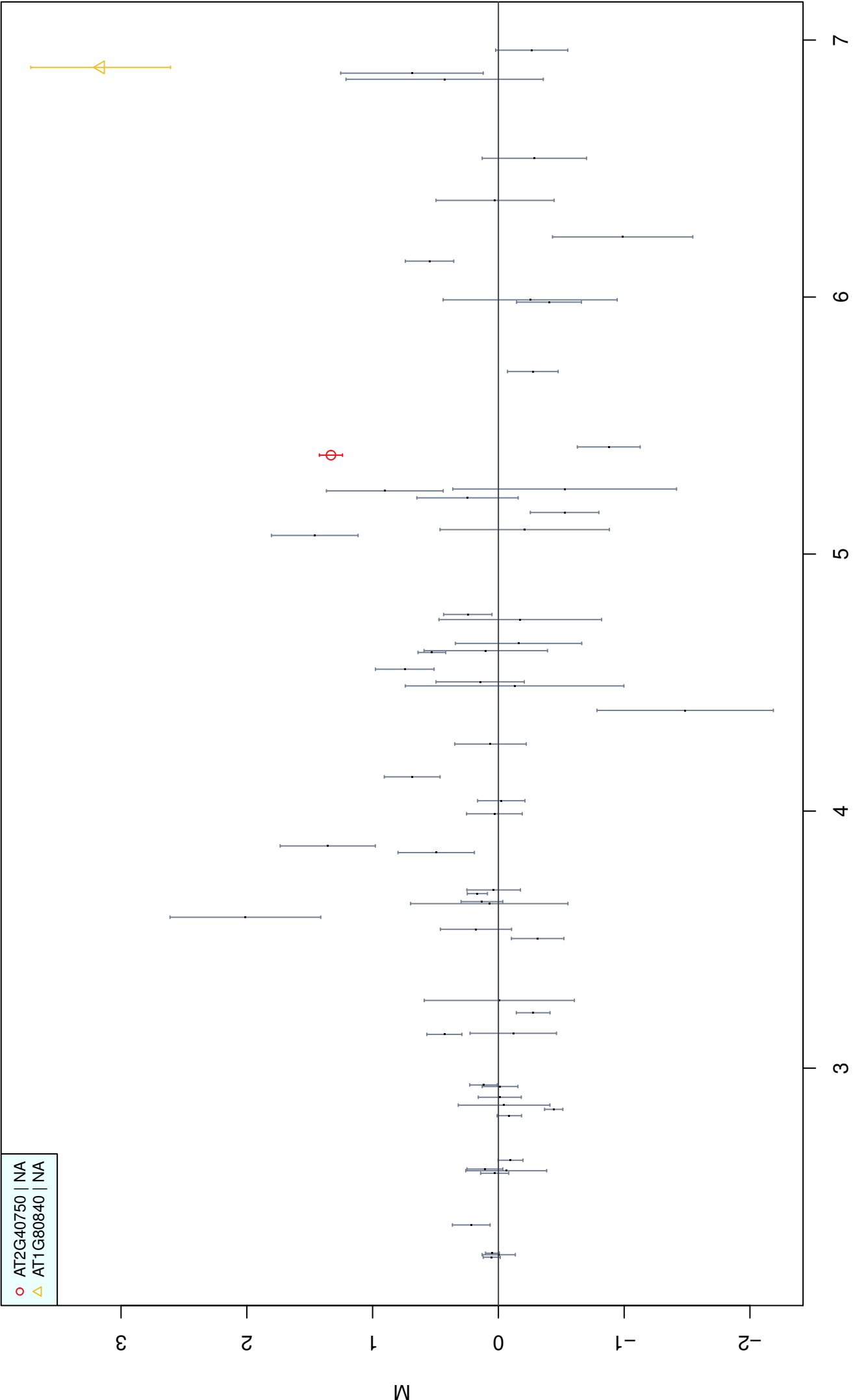

A  
Highlighted q<5%

Supplement: Figure S3 — MA plot (15 dpi syncytium vs. 5 dpi syncytium) for WRKY genes. (PDF) [file pone.0102360.s003.pdf]

Contrast: Syn vs control

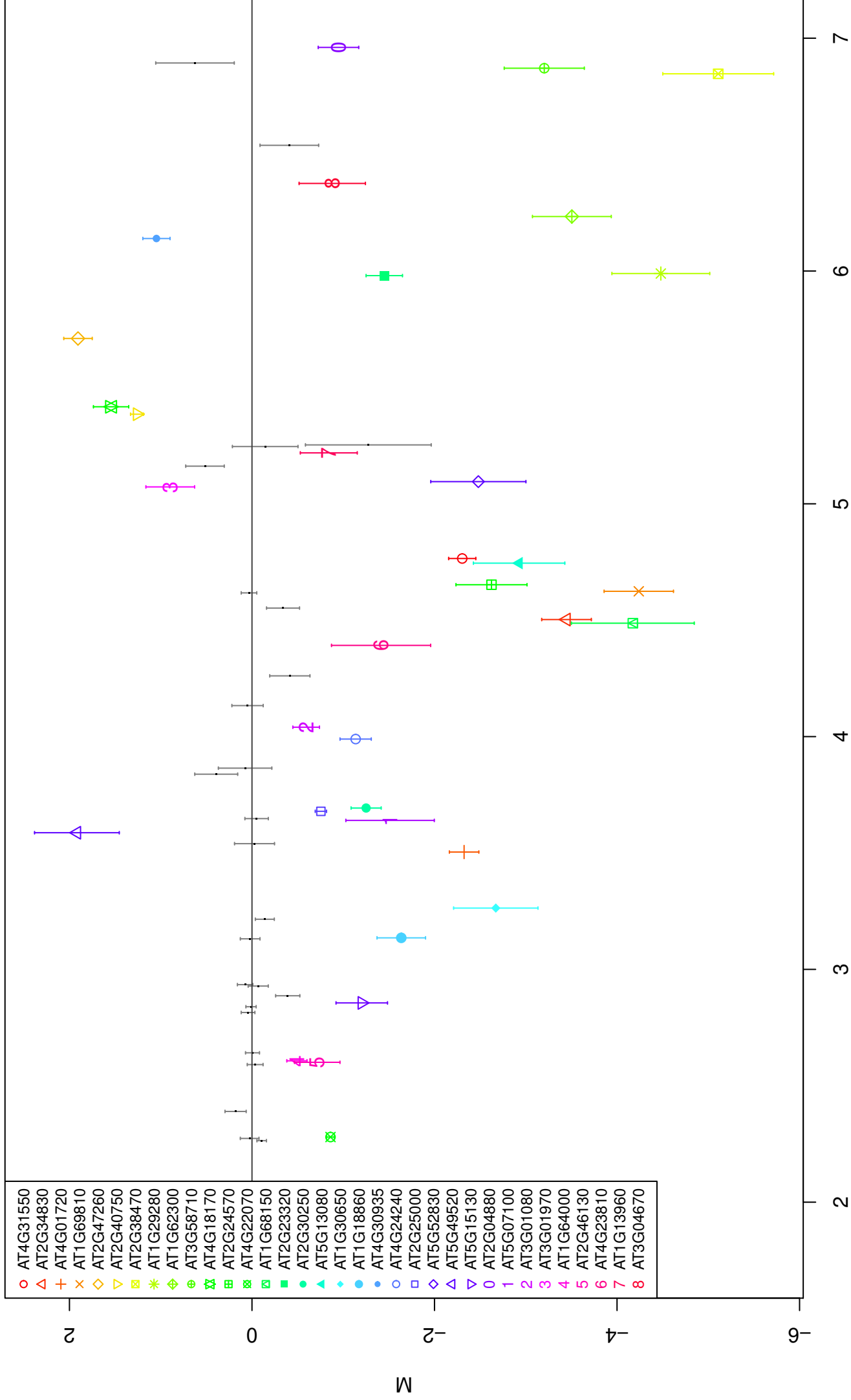

A  
Highlighted  $q < 5\%$

Supplement: Figure S4 — MA plot (syncytium vs. root) for WRKY genes. (PDF) [file pone.0102360.s004.pdf]
