# Supplementary material for: The Beet Cyst Nematode Heterodera schachtii Modulates the Expression of WRKY Transcription Factors in Syncytia to Favour Its Development in Arabidopsis Roots
Source: PLoS One. 2014 Jul 17;9(7):e102360. doi: 10.1371/journal.pone.0102360 (PMC4102525; doi:10.1371/journal.pone.0102360)
Supplement: Table S1 — Primers used in this work. (PDF) [file pone.0102360.s005.pdf]

Table S1: Primers used in this study

pMIOX5for_Eco	AGAGAATTCGAGGAAGATGAGACTGA
pMIOX5rev_Nco	ATATCCATGGCATCTTCCAAAAAAAAACAAAGT
cWRKY33forNco	TTCCCATGGCTGCTTCTTTTCTTAC
cWRKY33revBam	TTCCTTGGATCCTCAGGGCATAAAC
WRKY33RTfor	GTGTACAATGCCAGTTTGGATCA
WRKY33RTrev	GATGGTTGTGCACTTGTAGTAGC
WRKY33seq1	CTAGCTTCTCCAACCACAGGAG
WRKY33rseq2	CGTTGTCTGCACTACGATTCTC
WRKY33qRTfor	GAAGATTGTGGGAGTGAACCTGA
WRKY33qRTrev	ACTTGTAGTAGCTTCTTGGATTTGGG
q18Sfor	GGTGGTAACGGGTGACGGAGAAT
q18Srev	CGCCGACCGAAGGGACAAGCCGA
MKK4forNco2	AAAGCCATGGGACCGATTCAATCGCCTCCAG
MKK4Mfor	GATATGGACCCGTGTAATGACTCTGTTGGAACC
MKK4Mrev	GTCATTACACGGGTCCATATCCTGAGCCAAAGG
MKK4revBam	AGAGGATCCGCTATGTGGTTGGAGAAG
promWRKY17forEcoRI	TTTCGAATTCGCTGTTCTCAACTAAAGAC
promWRKY17revNcoI	AACGGCCATGGTGAGAAACCAGAGGAG
