# Supplementary material for: The Beet Cyst Nematode Heterodera schachtii Modulates the Expression of WRKY Transcription Factors in Syncytia to Favour Its Development in Arabidopsis Roots
Source: PLoS One. 2014 Jul 17;9(7):e102360. doi: 10.1371/journal.pone.0102360 (PMC4102525; doi:10.1371/journal.pone.0102360)
Supplement: File S1 — Online methods bioinformatic analysis. (PDF) [file pone.0102360.s006.pdf]

Additional file 1 – Online methods bioinformatic analysis

Microarray analysis

The eleven hybridizations were conducted by the German Resource Centre for Genome Research GmbH (now ATLAS Biolabs GmbH; both in Berlin, Germany) following the manufacturer's protocols (for details see [4]). Affymetrix CEL files were read into the R statistical analysis environment (www.r-project.org) using the *affy* package of the Bioconductor suite (www.bioconductor.org). As 10–40% of probe sets are affected by updated gene annotation, chips were processed with current TAIR v8 probe-set annotation [1]. Probe sequence specific 'background correction' [2] was performed using routines available in the Bioconductor *gcrma* package. Using the 'affinity' model, while 'MM' probes were employed for the determination of affinity parameters, only 'PM' probes were used for the probe-specific background correction. An inspection of exploratory pairwise scatter and 'MA' plots confirmed the need for inter-chip normalization. The thus required explicit normalization steps made a subtraction of the heuristic estimate for optical instrument background as offered in gcrma unnecessary. Defaults were used for all other *gcrma* parameters. As an examination of pairwise quantile-quantile plots showed only random fluctuations, inter-chip normalization could be achieved using quantile-quantile normalization [3]. See 'Low-level microarray analysis and diagnostic plots' section of the Online Supplement for diagnostic plots and figures [4]. After normalization, robust summaries of probe set signals were obtained for each gene using an iterative weighted least squares fit of a linear probe level model [5] through the *fitPLM* function of the Bioconductor package *affyPLM*. This process automatically identifies unreliable chip areas and correspondingly downweights outlier

probes. See Online Supplement for figures [4]. The normalized data on log2 scale were then fitted gene by gene with a linear model including hybridization batch effects, using the *lmFit* function [6] of the Bioconductor package *limma*. The result-tables also include *q*-values as indicators of significance of contrasts after correction for multiple testing controlling the False Discovery Rate [7]. For the statistical tests, individual gene variances have been moderated using an Empirical Bayes approach that draws strength from transferring variance characteristics from the set of all genes to the test for each individual gene [6]. Tests were restricted to a subset of 59 genes of the *WRKY* group. This considerably increases the statistical power of the testing procedure as it reduces the necessary correction for massive multiple testing.

Additional References

1. Dai MH, Wang PL, Boyd AD, Kostov G, Athey B, Jones EG, Bunney WE, Myers RM, Speed TP, Akil H *et al*: Evolving gene/transcript definitions significantly alter the interpretation of GeneChip data. *Nucleic Acids Res* 2005, 33(20).
2. Wu ZJ, Irizarry RA, Gentleman R, Martinez-Murillo F, Spencer F: A model-based background adjustment for oligonucleotide expression arrays. *J Am Stat Assoc* 2004, 99(468):909-917.
3. Bolstad BM, Irizarry RA, Astrand M, Speed TP: A comparison of normalization methods for high density oligonucleotide array data based on variance and bias. *Bioinformatics* 2003, 19(2):185-193.
4. Szakasits D, Heinen P, Wieczorek K, Hofmann J, Wagner F, Kreil DP, Sykacek P, Grundler FMW, Bohlmann H: The transcriptome of syncytia induced by the

cyst nematode Heterodera schachtii in Arabidopsis roots. *Plant J* 2009, 57(5):771-784.

5. Bolstad BM: Low Level Analysis of High-density Oligonucleotide Array Data: Background, Normalization and Summarization. Berkeley: University of California; 2004.

6. Smyth GK: Linear models and empirical bayes methods for assessing differential expression in microarray experiments. *Stat Appl Genet Mol Biol* 2004, 3:Article3.

7. Benjamini Y, Hochberg, Y.: Controlling the false discoveryrate: a practical and powerful approach to multiple testing. *J R Stat Soc Ser* 1995, B 57:289-300.8.
